# Cultural considerations in the classification of mental disorders: why and how in ICD-11

**DOI:** 10.1186/s12916-020-1493-4

**Published:** 2020-01-27

**Authors:** Oye Gureje, Roberto Lewis-Fernandez, Brian J. Hall, Geoffrey M. Reed

**Affiliations:** 1 WHO Collaborating Centre for Research and Training in Mental Health, Neuroscience and Substance Abuse, Department of Psychiatry, University of Ibadan, Ibadan, Nigeria; 2 Department of Psychiatry, Stellenbosch University, Stellenbosch, South Africa; 3 Department of Psychiatry, Columbia University Vagelos College of Physicians and Surgeons, New York, NY USA; 4 New York State Psychiatric Institute, New York, NY USA; 5 Department of Global Mental and Social Medicine, Harvard Medical School, Boston, MA USA; 6 Global and Community Mental Health Research Group, Department of Psychology, Faculty of Social Sciences, The University of Macau, Macao, SAR People’s Republic of China; 7 Department of Mental Health and Substance Abuse, World Health Organization, Geneva, Switzerland

**Keywords:** ICD-11, Culture, Classification, Mental disorders

## Background

The origins and manifestations of mental disorders are complex, reflecting biological, psychological, and socio-cultural influences [1]. Psychopathology cannot be explained on the basis of brain dysfunction alone. The environment in which psychopathology develops has a major impact, with cultural influences playing a particularly salient role. Culture is involved in conceptualizations of what constitutes normality and deviation from it; further, it influences coping schemas, help-seeking behaviors, as well as the expression and course of mental illness. Indeed, at higher levels of health systems, cultural factors affect social policies that protect individuals from risk of developing mental disorders or determine access to care. For health classifications to faithfully represent the interface between health encounters and health information [2], they need to reflect the broader cultural contexts in which illness is experienced. Such considerations informed the decision of the World Health Organization’s Department of Health and Substance Abuse to focus on the development of guidance for culture in the use of the Chapter on Mental and Behavioral Disorders in the 11th edition of the International Classification of Diseases (ICD-11).

## Main text

### Salience of culture

Although the impact of culture on psychopathology may now be more generally accepted, historical records contain many instances of biological reductionism. In the 1950s, it was possible for Carothers, the British colonial psychiatrist working in Africa, to attribute his inability to detect obsessions among his African subjects to “*poorly developed frontal lobes*”, a condition he considered the functional equivalent of a leucotomy [3]. These types of observations do not take into account the impact of the cultural context on psychopathology or the cultural biases that the observer brings to the cross-cultural encounter. Other types of misattributions can occur when non-homogenous constructs are grouped together as cultural entities, such as ‘developed’ and ‘developing’ countries or ‘white’ and ‘black’ racial groups. Such misattributions can be gradually corrected through the work of more culturally embedded researchers [4]. Nevertheless, the field may retain a bias for seeking explanations solely within the confines of the body for health conditions that are profoundly influenced by social disadvantage and perpetuated by culturally determined values and priorities.

### Culture in the ICD-11

International classification has the challenge of deciding on appropriate ways of reflecting the influence of culture on the pattern and presentation of mental disorders. A major focus of the 11th edition of the ICD is clinical utility [5], which requires a consideration of cultural factors that may be relevant to decision-making during the clinical encounter. A major goal is to provide a basis for discourse among patients, caregivers, health professionals, and policymakers. A “*common language*” [6] is important to facilitate communication and valid decision-making in mental health care. Careful delineation of the cultural issues in the context of a globally applicable diagnostic system can help the clinician make informed decisions about the patient’s condition and negotiate appropriate care, while retaining the ability to communicate the clinical condition to other providers within and outside the immediate cultural setting.

Guidance for considering culture when using the ICD-11 was developed by a panel of experts after extensive review of the literature and the relevant cultural formulations in the ICD-10 and the 5th edition of the Diagnostic and Statistical Manual [7]. This represents a pragmatic balance between the need for a global classificatory system that can facilitate reliable communication of clinical information across geographic and cultural boundaries while retaining the ability to be contextually and culturally relevant during the clinical encounter.

## Conclusion

The guidance for cultural considerations in ICD-11 should enhance the clinical utility of the constituent diagnostic constructs and help clinicians make informed decisions. However, culture is a complex phenomenon and its ramifications are protean. A truly culturally sensitive classification that reflects this complexity is difficult to achieve for global use. One way of enhancing cultural sensitivity is by ensuring that the process of constructing the parameters of what constitutes psychiatric ‘caseness’ taps into diverse cultural experiences through inclusive decision-making [8]. This is particularly so given that, currently, what constitutes a psychiatric disorder is not decided on the basis of immutable neuroscientific validating features, but rather on best expert judgment. The greater the breadth of cultural experiences informing that judgment, the more likely the classification will be able to serve as a truly global medium of clinical exchange of information.

## Data Availability

Not applicable.
